# Thermal Insulation and Fireproof Aerogel Composites for Automotive Batteries

**DOI:** 10.3390/gels11100791

**Published:** 2025-10-02

**Authors:** Xianbo Hou, Jia Chen, Xuelei Fang, Rongzhu Xia, Shaowei Zhu, Tao Liu, Keyu Zhu, Liming Chen

**Affiliations:** College of Aerospace Engineering, Chongqing University, Chongqing 400030, China; xianbo_hou@cqu.edu.cn (X.H.);

**Keywords:** aerogel composites, automobile battery, thermal management, fireproof

## Abstract

New energy vehicles face a critical challenge in balancing the thermal safety management of high-specific-energy battery systems with the simultaneous improvement of energy density. With the large-scale application of high-energy-density systems such as silicon-based anodes and solid-state batteries, their inherent thermal runaway risks pose severe challenges to battery thermal management systems (BTMS). Currently, the thermal insulation performance, temperature resistance, and fire protection capabilities of flame-retardant materials (e.g., foam cotton, fiber felts) used in automotive batteries are inadequate to meet the demands of intense combustion and high temperatures generated during thermal failure in high-energy-density batteries. Against this backdrop, thermal insulation and fireproof aerogel materials are emerging as a revolutionary solution for the next generation of power battery thermal protection systems. Leveraging their nanoporous structure’s exceptional thermal insulation properties (thermal conductivity of 0.013–0.018 W/(m·K) at room temperature) and extreme fire resistance (temperature resistance > 1100 °C/UL94 V-0 flame retardancy), aerogels are gaining prominence. This article provides a systematic review of thermal runaway phenomena in automotive batteries and corresponding protective measures. It highlights recent breakthroughs in the selection of material systems, optimization of preparation processes, and fiber–matrix composite technologies for automotive fireproof aerogel composites. The core engineering values of these materials, such as blocking thermal runaway propagation, reducing system weight, and improving volumetric efficiency, are quantitatively validated. Furthermore, the paper explores future research directions, including the development of low-cost aerogel composites and the design of organic–inorganic hybrid composite structures, aiming to provide a foundation and industrial pathway for the research and development of next-generation high-performance battery thermal management systems.

## 1. Introduction

As the core component of new energy vehicles, power batteries directly determine the overall performance, safety, and service life of the vehicle [1,2,3]. The current level of technological development in power batteries has become a critical factor driving the industrialization of new energy vehicles [4,5,6]. Among these, battery thermal safety remains a core challenge for the industry [7,8,9,10]. Lithium-ion batteries dominate the new energy vehicle market (with a market share exceeding 90%) due to their advantages such as high energy density (typically reaching 200–300 Wh/kg), long cycle life (>2000 cycles), and operational capability across a wide temperature range (−20 °C to 60 °C) [11,12,13,14,15]. However, factors such as battery aging, mechanical impact, high-temperature environments, thermal runaway, and overload use can potentially trigger fire accidents [7,16,17]. Under abnormal operating conditions, particularly during overcharging or overheating, a chain exothermic reaction occurs inside lithium-ion batteries, leading to thermal runaway and the release of a massive amount of thermal energy within milliseconds (with temperatures exceeding 800 °C) [18,19,20]. This results in risks of combustion and explosion. Statistics indicate that approximately 70% of spontaneous combustion incidents in new energy vehicles originate from internal battery insulation failure [21,22,23,24]. When an individual battery cell releases several megajoules of heat instantaneously due to internal or external short circuits, the temperature surges at a rate of hundreds of degrees Celsius per second [25,26,27,28,29]. The runaway battery triggers cascading thermal runaway in adjacent cells through heat conduction and flame jetting, ultimately leading to systemic failure of the battery module or even the entire pack. This can cause catastrophic fires or explosions [30,31,32,33,34].

Aerogel, as a three-dimensional nanoporous network material constructed from colloidal particles or polymers (porosity > 90%), demonstrates broad application prospects by virtue of its unique structural characteristics [35,36,37,38]. Its framework consists of nanoscale solid particles (typically 2–20 nm in diameter), endowing the material with ultra-low density (3–150 kg/m^3^), sub-micron pore size (<100 nm), ultra-high specific surface area (500–1200 m^2^/g), and ultra-low thermal conductivity (0.013–0.018 W/(m·K)) [39,40,41,42,43]. Since Kistler first synthesized silica aerogel in 1931 using sol–gel method combined with supercritical fluid drying technology, diverse aerogel systems including alumina, zirconia, polyimide (PI), and cellulose have been successively developed, with continuous optimization in mechanical properties, thermal insulation performance, and temperature resistance [44,45,46,47]. These characteristics grant aerogel exceptional fire resistance (withstanding flames >1000 °C), near-limit thermal insulation capability, and a combination of light weight and high strength, making it an ideal material for enhancing the safety of power batteries [48,49,50,51,52]. By compositing nano-aerogel with matrices such as glass fibers and ceramic fibers, flexible, lightweight (areal density <1.5 kg/m^2^), and highly thermally insulating aerogel composite materials have been successfully developed [53,54,55,56]. Such materials not only exhibit long-term service life of over 20 years, ultra-low thermal conductivity of approximately 0.02 W/(m·K), and UL94 V-0-level flame retardancy, but also demonstrate excellent compressive strength (>0.5 MPa) and resilience [57,58,59]. When applied in the power battery packs of new energy vehicles, these materials can form highly efficient thermal barriers, significantly enhancing the fire protection level of the battery system and providing core safeguards for driving and passenger safety.

Addressing the challenge of cascading thermal runaway in battery packs, the current mainstream solution involves implanting functional thermal insulation and fireproof layers between cells [7,60,61]. Such barrier layers can effectively block heat transfer from a runaway cell to adjacent batteries, thereby maximizing the containment of thermal propagation. Currently deployed thermal insulation materials for power batteries primarily include polymer foams [62,63,64,65,66], inorganic fiber mats (glass wool/high-silica wool) [67,68], vacuum insulation panels [69,70,71,72], and aerogel-based composites [73,74,75,76,77,78,79,80,81]. Among these, aerogel insulation sheets placed between cells leverage their nanoporous network structure to delay thermal runaway propagation, far exceeding the protective endurance of traditional materials. Notably, aerogel, as a porous solid with gas as the continuous phase, demonstrates disruptive advantages in power battery applications: its thermal conductivity is only one-third to one-fifth that of conventional materials, allowing for a 50–80% reduction in thickness under equivalent insulation requirements, operates stably across a temperature range of −200 °C to 650 °C, and contains no volatile toxic substances. Given the stringent demands of power batteries for light weight and spatial efficiency, aerogel pads have been widely recognized as the thinnest (0.5–3 mm) and most effective thermal management solution. Experimental evidence confirms that they not only block thermal runaway propagation but also reduce performance degradation in low-temperature environments, while saving valuable space within the battery pack. With global annual production of new energy vehicles exceeding 30 million units, the demand for aerogel is experiencing exponential growth.

Against the backdrop of the rapid development of the new energy vehicle industry, thermal safety management of lithium-ion battery systems has become a key focus of technological breakthroughs. As a new generation of nanoscale thermal insulation material, aerogel composites are gradually emerging as a benchmark solution for fire prevention and thermal insulation in power batteries, owing to their exceptional resistance to extreme high temperatures, ultra-low thermal conductivity, lightweight properties, and environmental advantages. This article systematically analyzes the mechanism of thermal runaway in automotive batteries and the evolution of protection technologies, with a focused exposition on the structural characteristics of aerogel insulation materials—such as dual hydrophobic–oleophobic surface designs that resist electrolyte corrosion—as well as their performance advantages and their mechanism for suppressing thermal propagation at the module and pack levels. By comparing key parameters of traditional insulation materials, it is confirmed that aerogel composites represent a revolutionary breakthrough in terms of insulation efficiency, thickness reduction, flame retardancy rating, and long-term stability. As the large-scale production costs of aerogels continue to decline, their integrated innovation with technologies such as phase change materials and fire-resistant coatings will play a central protective role in next-generation high-voltage platform battery packs.

## 2. Thermal Runaway Phenomenon and Thermal Protection Measures of Automotive Batteries

### 2.1. Thermal Runaway of Automotive Batteries

Thermal runaway refers to an uncontrollable process where the temperature of a battery rises sharply due to a chain reaction of exothermic reactions within the battery, leading to fire or explosion. The main causes include short circuits, impacts, high temperatures, and excessive charging and discharging [82]. Phenomena such as swelling, bulging, smoking, ignition, and combustion occur sequentially [82,83]. During this process, the electrical and chemical energy within the battery is converted into a significant amount of thermal energy [84,85]. According to the principles of heat transfer, this heat is spontaneously transferred to adjacent batteries or electrical components, inducing thermal runaway in surrounding cells [86,87]. Ultimately, this can result in the entire battery system catching fire or even the whole vehicle burning.

**Figure 1:**
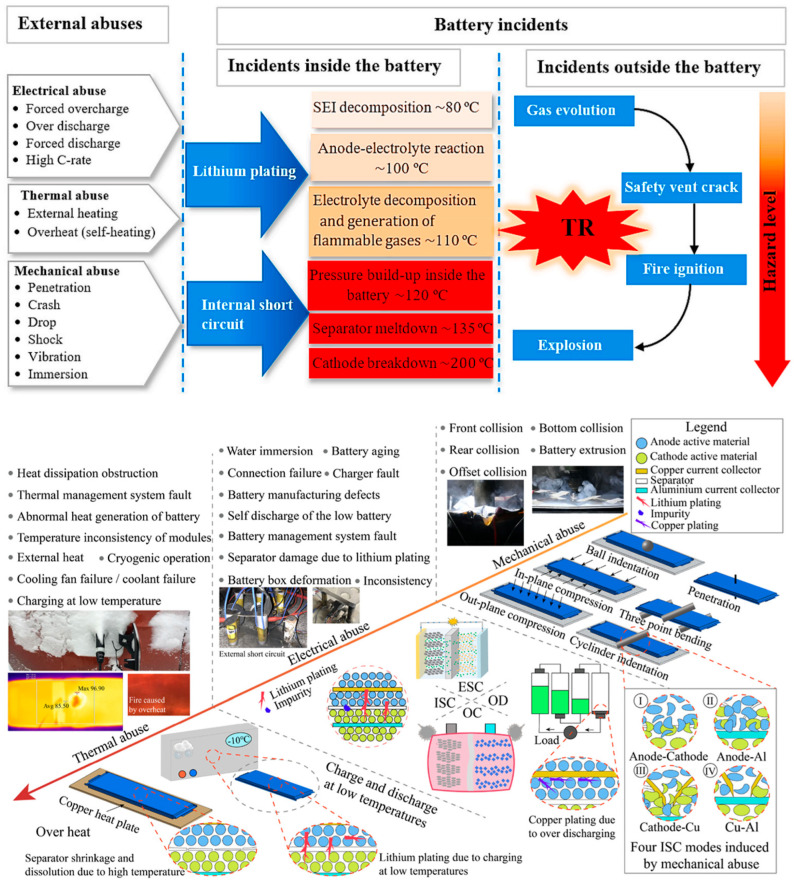
Summary of the abuse of automotive battery packs [88] and the detailed fault causes of lithium-ion batteries under mechanical, electrical and thermal abuse conditions (from the real world to the laboratory) [89]. Reproduced with permission from ref. [88]. Copyright 2020 Elsevier. Reproduced with permission from ref. [89]. Copyright 2023 Elsevier.

Battery thermal runaway is primarily categorized into three types as shown in Figure 1—mechanical abuse, electrical abuse, and thermal abuse—mainly caused by the following reasons [88,89]: (1) Internal short circuit: A short circuit may occur between the positive and negative electrodes inside the battery, leading to a sharp increase in current and generating substantial heat. This situation is often due to defects or damage in the battery’s internal structure. (2) Overcharging or over-discharging: Overcharging or over-discharging can intensify chemical reactions inside the battery, producing excessive heat. This may cause a rapid rise in the battery’s internal temperature, eventually leading to thermal runaway. (3) High-temperature environment: When a battery operates in a high-temperature environment, its temperature is prone to increase, accelerating chemical reactions and potentially triggering thermal runaway. Therefore, exposure to high-temperature environments should be avoided during use. (4) Mechanical damage: When a battery suffers mechanical damage, such as impact or compression, its internal structure may be compromised, leading to thermal runaway [90,91,92,93]. Additionally, the battery’s own condition is one of the critical factors contributing to thermal runaway. During normal charge–discharge cycles, batteries age with use, manifesting phenomena such as lithium deposition, damage to electrode structures, phase changes in electrode materials, and decomposition of active materials and electrolytes. These result in capacity decay, increased internal resistance, and reduced safety performance of the battery system. Undesirable side reactions, such as the formation of metallic dendrites, can easily pierce the separator, causing local internal short circuits [94,95,96].

Research indicates that when the ambient temperature does not exceed 80 °C, the battery can charge and discharge normally. However, when the battery temperature exceeds 80 °C, its internal materials begin to decompose, battery performance declines, and it enters a failure phase. As the temperature continues to rise, the battery separator fails, causing the positive and negative electrode materials to come into contact. This results in an internal short circuit, generating a substantial amount of heat. This leads to a continuous increase in the internal temperature of the battery cell, thereby inducing thermal runaway. Notably, once a single battery cell experiences thermal runaway, adjacent cells are also susceptible to consecutive thermal runaway due to the effects of heat diffusion. This leads to the propagation of thermal runaway, known as thermal diffusion, ultimately causing the battery to catch fire. In severe cases, explosions may even occur. In recent years, frequent safety incidents in electric vehicles caused by thermal runaway of lithium-ion batteries have severely hindered the commercial development of lithium-ion batteries in the electric vehicle industry [97,98].

### 2.2. Thermal Protection of Automotive Batteries

Thermal runaway propagation in batteries occurs rapidly, characterized by large flames and highly destructive forces. In practical battery packs, thermal runaway can easily cause numerous individual cells to overheat. The rapid diffusion of heat leads to large-scale thermal runaway, resulting in the entire battery system or even the entire vehicle igniting and burning. Preventing thermal runaway and ensuring the safe operation of power batteries have consistently been key focuses of research and development for new energy vehicle manufacturers [98].

**Figure 2:**
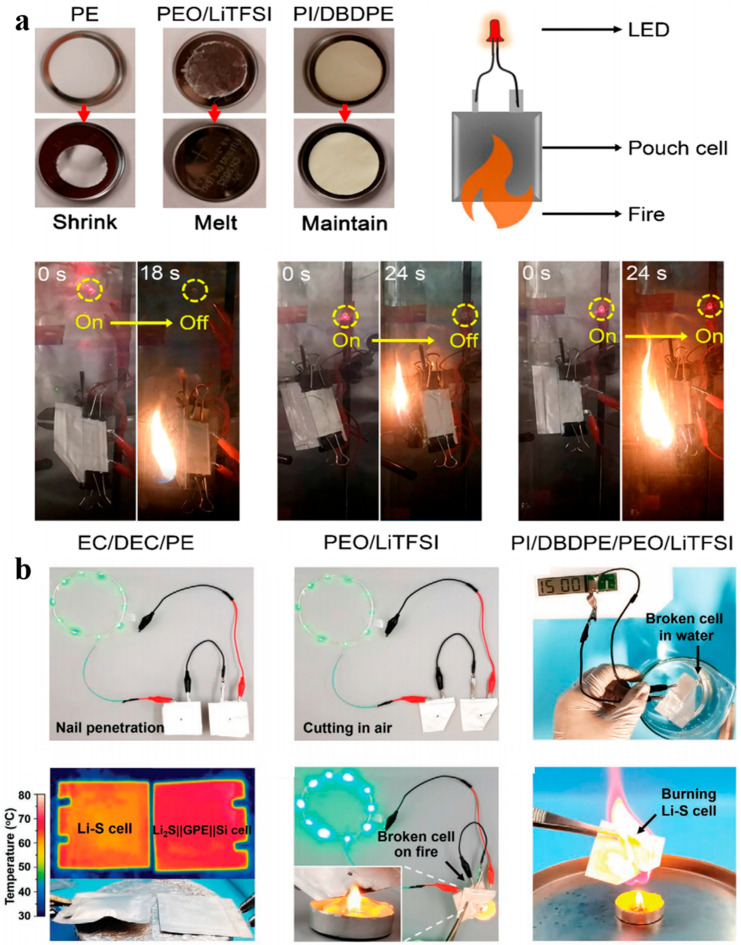
Enhancing battery safety through the development of novel fire-resistant electrolytes, (**a**) Battery flame abuse test; (**b**) Safety assessment of quasi-solid-state batteries [103,104]. Reproduced with permission from ref. [103]. Copyright 2022 Wiley-VCH. Reproduced with permission from ref. [104]. Copyright 2020 American Chemical Society.

Current solutions primarily concentrate on two approaches. The first involves monitoring the operational status of batteries through a combined hardware and software vehicle thermal runaway protection system, aiming to prevent the occurrence of thermal runaway or delay its propagation as much as possible, thereby enhancing the safety of the battery system. The second method utilizes materials with excellent insulation properties as battery electrolytes, separators, and fire-resistant insulation materials to cut off heat transfer, preventing and delaying the onset of thermal runaway, as shown in Figure 2a,b [99,100,101,102,103,104]. For example, several studies have focused on enhancing battery safety through electrolyte and separator modifications. Meng et al. developed a quasi-solid-state battery incorporating a fire-resistant gel electrolyte to improve safety and energy density [103]. Cui et al. designed an ultra-lightweight polymer-based solid-state electrolyte with inherent flame retardancy, enabling stable operation of pouch cells even under direct flame exposure [104]. Meanwhile, Sundaram et al. developed high-concentration aqueous electrolytes (such as “water-soluble salt”-type electrolytes), which demonstrated a larger electrochemical (voltage) window and battery safety [105]. Although these electrolyte- and separator-based strategies contribute to internal safety, they remain complementary to external thermal barriers. In contrast, aerogel-based materials offer a more direct and physically robust solution for blocking heat propagation, making them particularly suitable as core interlayer thermal barriers in high-energy-density battery systems.

**Figure 3:**
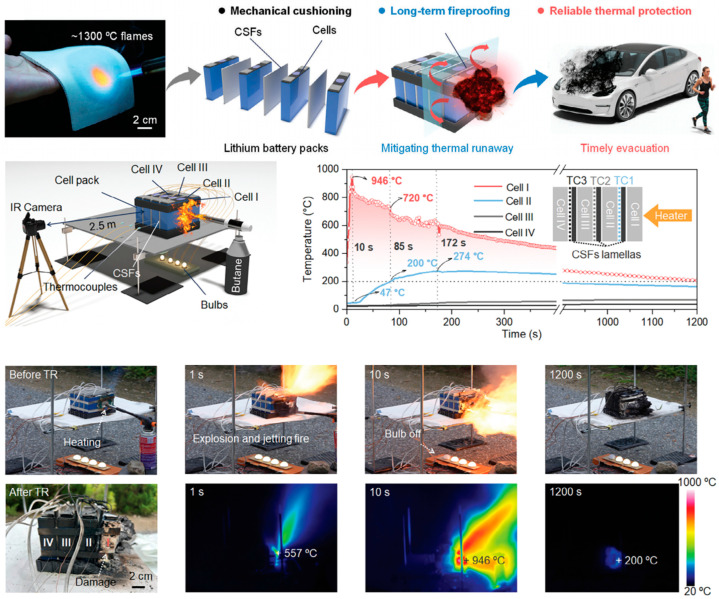
Multi-scale filler synergistic ceramization strategy to manufacture silicone foam nanocomposites with flexibility and long-term fire resistance [118]. Reproduced with permission from ref. [118]. Copyright 2025 Wiley-VCH.

The use of thermal insulation materials to protect batteries has become the preferred solution for automakers addressing battery thermal runaway. Various insulation materials are commonly employed in electric vehicle battery packs, including composites such as aerogels and aerogel coatings [106,107,108,109,110], polyurethane foams [111,112,113], glass fibers [114,115], and PI films [116,117]. For instance, Chen et al. proposed a multi-scale filler synergistic ceramization strategy to manufacture silicone foam nanocomposites with flexibility and long-term fire resistance, as shown in Figure 3 [118]. This foam not only maintains mechanical flexibility and elasticity within a temperature range of −60 °C to 210 °C (exhibiting a residual strain of approximately 2% after 1000 cycles) but also demonstrates exceptional long-term (>30 min) thermal insulation performance when exposed to an oxidative environment at around 1300 °C, owing to its ability to form a gradient robust porous ceramic structure. Chen et al. developed a novel battery separator with high safety. By adding a trace amount of surfactant sodium dodecyl sulfate (SDS) to the béchamel (BM) slurry, they successfully achieved uniform wetting of polyvinylidene fluoride/polyetherimide (PVDF/PEI) coaxial electrospun fiber membranes, significantly enhancing the membrane’s flame retardancy and thermal stability at high temperatures [119].

These insulation materials play a critical role in electric vehicles, effectively safeguarding the temperature stability of the battery pack, extending its service life, and enhancing its overall safety performance. The insulation boards inside the battery pack are thermal protection devices placed between individual cells. They can effectively delay or block the propagation of thermal runaway from a single cell to the entire battery system. These boards must possess properties such as flame retardancy, high-temperature resistance, low thermal conductivity, no generation of toxic gases, light weight, and thinness. Aerogel insulation composites not only exhibit these characteristics but also offer excellent safety performance. They are recognized as the thinnest and most efficient insulation material currently applied in power batteries for new energy vehicles.

## 3. Heat-Insulating and Fire-Resistant Aerogel Composite Materials

**Figure 4:**
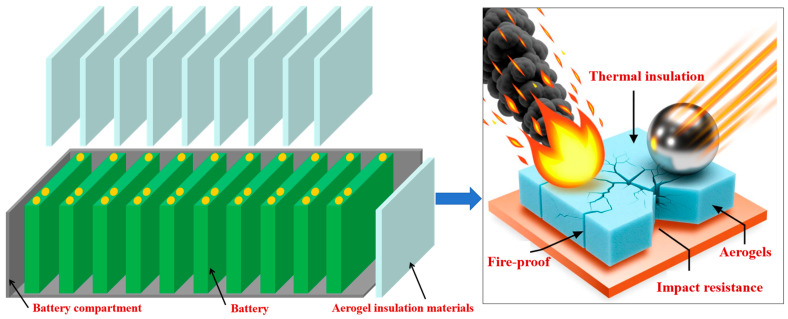
Battery structure diagram and schematic diagram of aerogel for heat insulation, fire prevention and impact resistance.

The heat insulation and fireproof materials for car batteries usually include foams, vacuum insulation boards, rock wool, fireproof cotton, inorganic aerogels, organic aerogels and aerogel composites, as shown in Table 1. The foam features a porous structure. In this structure, heat conduction is effectively blocked as air is sealed within the pores. The vacuum insulation panel eliminates internal gas convection and conduction by evacuating the air and further blocks radiation and solid heat transfer. In comparison, aerogels possess characteristics such as thermal insulation, flame retardancy, and ease of processing. As an ideal thermal insulation and flame-retardant material, they are becoming the preferred choice for automotive manufacturers as shown in Figure 4. The application of aerogel insulation materials in new energy vehicle power batteries is mainly reflected in the following aspects: (1) Excessively high internal battery temperatures can shorten battery life and reduce safety. By incorporating aerogel insulation materials into battery modules, the internal temperature of the battery can be effectively reduced, thereby mitigating the adverse effects of excessively low external temperatures on battery charging and discharging efficiency and prolonged charging times. This reduction in energy loss from internal reactions enhances battery efficiency. (2) The excellent insulation properties of aerogels can prevent safety hazards such as internal short circuits in batteries. Additionally, their stable chemical properties and good weather resistance can extend battery service life. (3) Aerogel insulation materials are characterized by low density. While providing insulation performance, they effectively increase the energy density of the battery. The high porosity of aerogel insulation materials enables functions such as sound absorption, noise reduction, and impact resistance as shown in Figure 4. When applied to vehicle body structures, they enhance the comfort of the riding environment, are waterproof and breathable, effectively inhibit mold growth, and improve interior air quality. (4) Aerogel insulation sheets exhibit good plasticity and can be shaped arbitrarily. They can be attached to interlayers in the form of irregular components, reducing heat conduction between battery packs and lowering the risk of sudden temperature spikes during thermal runaway, thereby providing a certain degree of personnel safety.

Aerogel insulation materials can significantly improve the heat resistance of battery modules. The application of aerogels in the thermal protection of power batteries can effectively prevent thermal diffusion caused by battery thermal runaway. Using aerogel insulation materials to isolate individual battery cells one by one can effectively block heat transfer between cells, prevent the domino effect triggered by thermal runaway in some cells within the battery pack, effectively delay or stop heat diffusion, provide sufficient escape time for vehicle occupants, and avoid personal injury. Therefore, aerogel insulation sheets have become an indispensable new material in the field of electric vehicle manufacturing and are gradually becoming an important design standard in the field of power battery safety protection. Simultaneously, aerogel materials used for thermal insulation and fire protection in automotive batteries must meet several stringent requirements: First, they must have extremely low thermal conductivity (generally ≤ 0.02 W/m·K) and withstand ultra-high temperatures (capable of enduring temperatures ranging from 650 °C to over 1200 °C) to effectively prevent high-temperature transfer during battery cell thermal runaway. Second, they must be lightweight and mechanically stable, maintaining structural integrity during the expansion and contraction of battery charging and discharging, and avoiding wrinkling or lithium plating at the battery cell interface. They must also exhibit excellent hydrophobicity (hydrophobicity ≥ 98%) and long-term weather resistance (service life ≥ 10 years) to address humid environments and aging issues. They must meet Class A fire protection standards and achieve effective thermal insulation within a certain thickness (generally 1–3 mm), providing at least 5 min (with a future target of 120 min) of escape time for personnel. This paper reviews high-temperature-resistant aerogels and their composite materials that are expected to be used for thermal insulation and fire protection in automotive batteries.

### 3.1. Organic Aerogels and Their Composite Materials Are Used for Protecting Against Battery Thermal Runaway

Organic aerogels and their composite materials offer distinct advantages owing to their cross-linked polymerization from multiple monomers, enabling tailored physicochemical properties through monomer selection [120,121,122,123]. They possess a unique three-dimensional nanoporous structure, ultra-high specific surface area, and robust mechanical properties, demonstrating revolutionary application potential in the field of new energy batteries. Current research focuses on polymer aerogel systems such as PI, cellulose, and polybenzoxazole (PBO), alongside the development of organic–inorganic hybrid materials compounded with high-temperature-resistant inorganic materials (e.g., carbon, silica nanoparticles, MXene, etc.). These hybrids maintain structural integrity even under extreme environmental conditions. Meanwhile, without effective hydrophobicity, the structure of aerogel will fail due to moisture absorption in a short time. Excellent hydrophobicity is the key to ensuring the stability of its thermal insulation, sound insulation, and other properties throughout the entire lifecycle of the product. The surface hydrophobicity of organic aerogel is also adjustable, and its surface energy can be precisely modified through the selection of precursor molecules (such as the introduction of functional groups such as fluorine, silane or amino) and subsequent treatment (such as pyrolysis, chemical vapor deposition, plasma treatment). Its inherent high porosity, huge specific surface area and complex network structure can significantly amplify the wetting effect brought by surface chemistry to build a rough surface, making the hydrophobicity of organic aerogel significantly improved.

**Figure 5:**
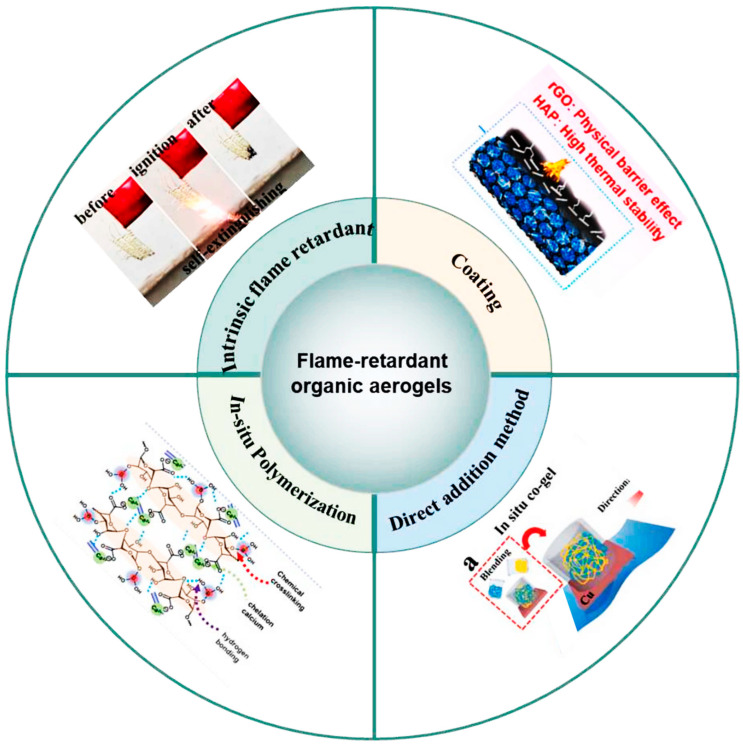
The flame-retardant strategies of flame-retardant organic aerogels [124]. Reproduced with permission from ref. [124]. Copyright 2024 Elsevier.

**Figure 6:**
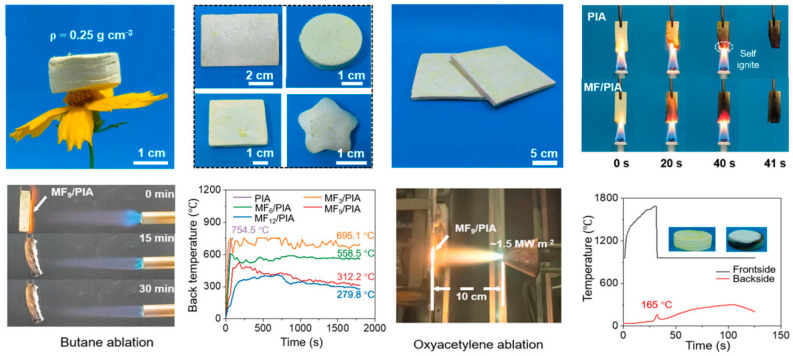
Lightweight mullite fiber/polyimide aerogel composites with superior ablation resistance [125]. Reproduced with permission from ref. [125]. Copyright 2025 Elsevier.

In terms of high-temperature resistance and flame retardancy, organic aerogel materials primarily achieve thermal insulation and fireproofing functions through molecular design and composite strategies [124], as shown in Figure 5. For instance, Wang et al. constructed mullite fiber-reinforced PI aerogel (MF/PIA) composites with a density of only 0.25 g cm^−3^ and a porosity of 90% using an environmentally friendly process involving atmospheric impregnation and drying, as shown in Figure 6 [125]. Silane coupling agents simultaneously reduced the surface energy of the wet gel (26.65 mN m^−1^) and strengthened the fiber–matrix interface, drastically reducing volume shrinkage from 61.3% to 27.9%. Under high heat flux (1.5 MW m^−2^ oxyacetylene flame), the material spontaneously formed a gradient structure comprising a “dense ceramic layer/functional transition layer/porous insulation layer” within 30 s, with a linear ablation rate as low as 6.8 μm s^−1^ and a backside temperature of merely 165 °C. Cao et al. prepared a series of methylguanidine-phenolic aerogels (MPA) via the copolymerization of methylguanidine and phenols during the sol–gel process [126]. Methylguanidine significantly enhanced the flame retardancy of the phenolic aerogel, with the modified phenolic aerogel containing 40 wt% methylguanidine achieving a limiting oxygen index (LOI) value of 32.7%. Han et al. utilized natural biomass and mineral nanostructures to fabricate fully natural wood aerogels with excellent thermal insulation and fire resistance. The addition of natural clay nanosheets substantially improved the aerogel’s fire resistance, enabling it to withstand high-temperature flames of 1300 °C for at least 20 min without burn-through.

**Figure 7:**
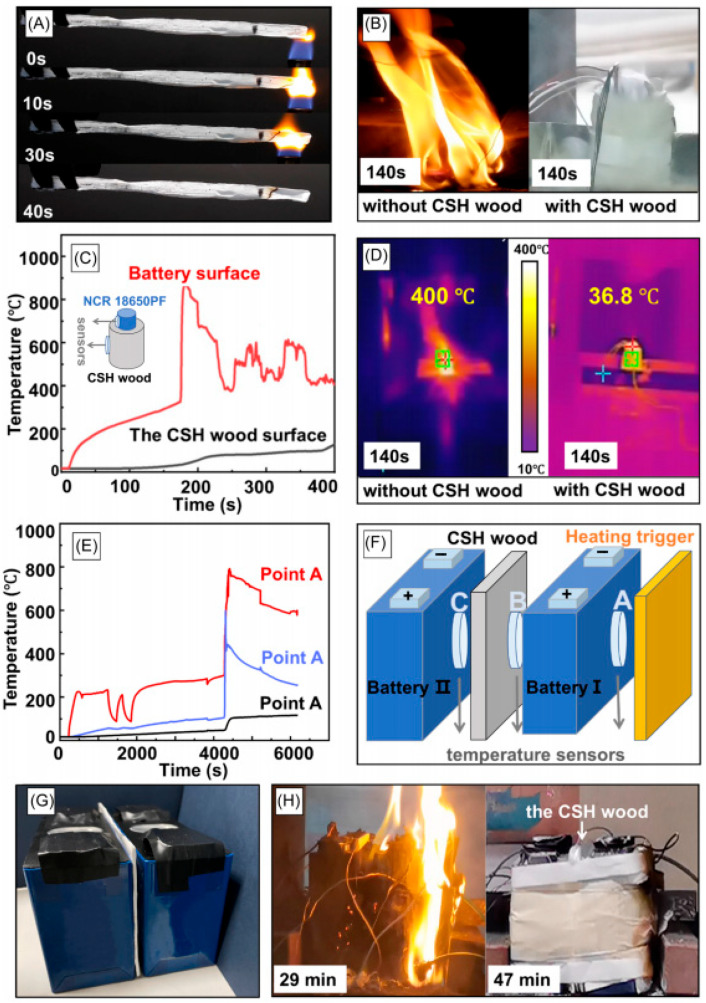
Fire retardancy of calcium silicate hydrate (CSH) wood in battery packages [131]. (**A**) Horizontal combustion test of CSH wood. (**B**) Temperature changes when a single lithium battery with CSH wood is exploded. (**C**) Infrared images of lithium batteries without and with CSH wood; the inset in the figure shows the placement diagram of the temperature sensor. (**D**) Photos of lithium batteries without and with CSH wood. (**E**) Temperature changes when two lithium battery packs with CSH wood are exploded. (**F**) Placement position of temperature sensors in the battery pack. (**G**) Two battery packs separated by 2 millimeters thick CSH wood. (**H**) Two battery packs with and without CSH wood. Reproduced with permission from ref. [131]. Copyright 2025 Wiley-VCH.

Wang et al. designed fully biomass-derived aerogels based on renewable pig gelatin (PG) and phytic acid sodium salt (PA) using a green freeze-drying method [127]. Compared to commercial polyurethane foam, these bio-based aerogels exhibited extremely low combustibility and superior smoke suppression properties, with an LOI as high as 50.1% and total smoke release reduced from 213 to 13.5 m^2^. Yu et al. employed a dual-template (ice and bubble) strategy to prepare ultra-lightweight, superelastic aerogels inspired by stress-dissipating dome structures, featuring hierarchical pore architectures [128]. Engineered microbubbles formed macropores (≈100 µm) via an improved “Tessari method,” while ice templating introduced aligned pores several micrometers in size during freeze-drying. Flame retardancy was achieved through potassium salt-mediated catalytic carbonation, reducing the peak heat release rate by 54% and imparting self-extinguishing behavior. Scalable production using commercial compressed air foaming systems and low-cost raw materials further highlighted industrial feasibility. Qian et al. developed PBO aerogels based on nanofiber physical entanglement and chemical cross-linking, achieving low density (3.6–15.7 mg cm^−3^), high porosity (98.9–99.7%), and high specific surface area (155.4 m^2^ g^−1^) [129]. The aerogel also exhibited excellent thermal stability, including high decomposition temperature (680 °C), long-term service temperature (350 °C), and outstanding fire resistance, achieving a V-0 rating in UL-94 tests, an LOI of 52.8%, and resistance to ignition under simulated real-fire conditions. When compounded with fumed silica, the aerogel withstood temperatures up to 1000 °C while providing superior thermal insulation. Liu et al. adopted an in situ skeleton encapsulation growth (ISEG) strategy to prepare PI aerogels (PIA) with binary organic–inorganic skeleton networks by in situ growth of polymethylsilsesquioxane (PMSQ) nanoparticles on the aerogel matrix network [130]. The linear shrinkage rate at 300 °C was only 1.11%. The robust skeleton of PIA-S18 ensured exceptional dimensional stability and enhanced mechanical properties even after extreme thermal shock cycles (ΔT = 496 °C), alongside resistance to burning under ultra-high-temperature flames (≈1200 °C). Du et al. developed an effective method for constructing hierarchical nanocomposite materials through the self-assembly of calcium silicate hydrates (CSH) and polyvinyl alcohol polymer chains. This multifunctional protective material, CSH wood, exhibits unprecedented lightweight characteristics (0.018 g per cubic centimeter), high stiffness (axial 204 megapascals), a negative Poisson ratio (−0.15), excellent toughness (6.67 × 10^5^ joules per cubic meter), excellent thermal insulation performance (radial 0.0204 watts per meter·kelvin), and excellent flame retardancy (UL94-V0 grade), as shown in Figure 7 [131].

**Figure 8:**
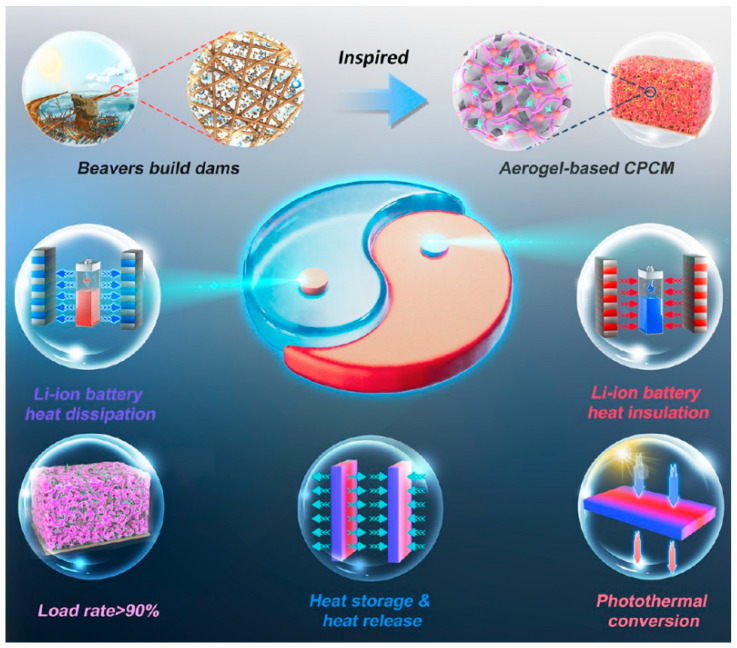
Dual-network aerogel-based composite PCM (CPCM) designed for the all-weather thermal control of Li-ion batteries [75]. Reproduced with permission from ref. [75]. Copyright 2025 American Chemical Society.

Bionic design further optimized flame-retardant mechanisms. For example, Li et al. developed a dual-network aerogel composite phase change material inspired by beaver dam construction, effectively regulating the thermal behavior of lithium-ion batteries, as shown in Figure 8 [75]. Through synergistic outer-layer carbonization for oxygen barrier and inner-layer phase change for heat absorption, the peak heat release was reduced by 67%, and ignition time was extended to 3.2 times that of conventional materials. Under high-temperature conditions, it ensured the battery’s maximum operating temperature remained below 55 °C; under low-temperature conditions, it maintained the battery above 10 °C for 30–40 min. Additionally, it preheated the battery, enhancing functionality in cold environments. However, challenges such as high costs, resilience degradation due to long-term thermal aging, and hydrophobic performance deterioration in humid environments remain bottlenecks for large-scale application.

**Figure 9:**
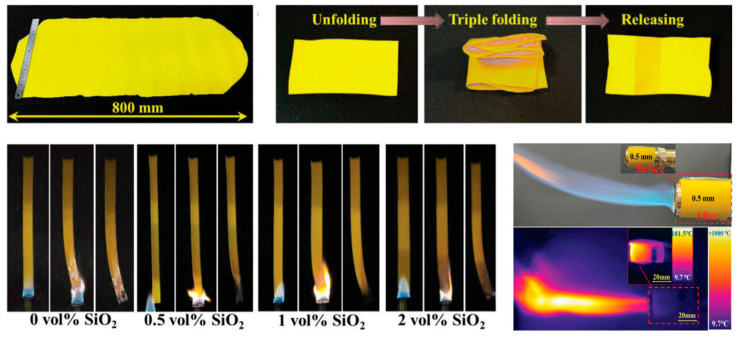
Photographs of polyimide aerogel composite (PIAC) and characterization of its thermal insulation and flame retardancy properties [123]. Reproduced with permission from ref. [123]. Copyright 2025 Wiley-VCH.

Among various types of organic aerogels, PI aerogels demonstrate significant technical advantages in automotive battery protection, with breakthroughs stemming from unique material design and preparation processes. While traditional inorganic aerogels excel in thermal insulation, their brittleness and poor mechanical shock resistance make them unsuitable for complex operating conditions in new energy vehicles. In contrast, PI aerogels achieve a balance of rigidity and flexibility through a dual-crosslinked structure combining flexible polymer chains and rigid inorganic networks. Compared to traditional silica aerogels, PI-based composite aerogels (e.g., PI@SiO_2_) incorporate organic polymer skeletons (e.g., PI) and inorganic nanoparticles (e.g., hydroxyapatite, silica), not only retaining the inherently ultra-low thermal conductivity (as low as 0.015–0.0357 W/(m·K)) but also significantly enhancing mechanical strength and high-temperature stability. For example, dual-crosslinked PI aerogel composites (PIAC) exhibit high flexibility, increasing tensile strength to 8.5 MPa and fracture toughness by 47.8 times. They remain stable across extreme temperatures from −110 °C to 500 °C, simultaneously exhibiting V-0 flame retardancy, as shown in Figure 9 [123], effectively resisting high-temperature impacts during battery thermal runaway and delaying heat transfer to adjacent cells or modules.

Additionally, innovations in preparation processes have reduced production costs and energy consumption, making them more suitable for mass production. In battery pack design, such materials can be fabricated into ultra-thin insulation layers (only 1–3 mm thick), reducing vehicle weight while optimizing battery performance through precise thermal management—improving low-temperature performance (e.g., increasing winter range by 15%) and high-temperature safety (e.g., blocking thermal runaway propagation). Their porous structure also absorbs mechanical impacts and noise, further enhancing vehicle safety and comfort. Future integration with advanced material technologies will further expand their application potential in new energy vehicle battery protection, positioning them as core materials driving industry safety standards.

From a practical perspective in new energy batteries, aerogels have evolved from single-function thermal insulation materials to multifunctional integrated platforms. Regarding thermal safety, Elison company’s nanocomposite aerogels achieve thermal conductivity as low as 0.012 W/(m·K), successfully controlling temperature differences within battery modules to within 2 °C. Their hydrophobicity increased to 150°, addressing failure issues in humid environments. CATL’s (Contemporary Amperex Technology Co. Limited, Fujian, China) patented “sandwich structure” battery functional layer combines aerogels with phase change materials (latent heat 180–220 J/g), reducing the positive electrode temperature rise rate by over 60% with less than a 5% increase in surface resistance, at a cost controlled below USD 0.2/m^2^. In electrode/separator innovation, graphene composite aerogels suppress organic electrode dissolution through three-dimensional confinement effects, significantly enhancing rate performance; cellulose aerogel separators exhibit thermal shrinkage <5% at 120 °C and effectively delay dendrite growth. Additionally, phase change composite aerogels expand temperature adaptation boundaries. Li et al.’s biomimetic materials combine high phase change enthalpy (178 J/g) with photothermal conversion capabilities, maintaining thermal balance for battery packs across a wide temperature range (−40 °C to 120 °C) and reducing peak temperatures by 12 °C under high-temperature conditions [75]. Low-cost preparation methods are key to promoting industrialization. Atmospheric drying processes largely replace energy-intensive supercritical techniques. Peng et al. developed a low-carbon thermal insulation aerogel through synergistic carbonization design and green atmospheric drying technology. Unlike traditional complex freeze-drying or hazardous solvent displacement methods, this technology combines thermally responsive gel fixation and mechanically assisted air templating to achieve green atmospheric drying conversion of aqueous foam hydrogels into porous aerogels. Researchers constructed a robust organic–inorganic composite network structure, endowing the aerogel with excellent fireproofing and mechanical properties: rapid self-extinguishment (LOI 50%), low heat/smoke hazards (30 kW/m^2^/1.6 m^2^), exceptional fire resistance (blocking 75.5% of heat from 1300 °C flames), and maintaining 93% structural/mechanical durability in harsh environments including high-temperature water, strong acids and alkalis, and various chemicals [132]. Creative utilization of biomass raw materials is even more disruptive. The economic inflection point for large-scale production has emerged—compressing gelation time and reducing drying energy consumption drive down the global average price of aerogel materials. Future drying technologies may exhibit “dual-track coexistence”: atmospheric processes dominate civilian applications, while supercritical processes retain high-end applications such as aerospace.

From current research, it can be concluded that future studies on organic aerogel materials need to focus on the following directions to break through bottlenecks: Multi-scale flame retardant synergy: Develop gradient flame retardant systems at molecular–nano–macro scales, aiming to maintain thermal conductivity below 0.03 W/(m·K) under 1200 °C flame impact, drawing on organic–inorganic hybrid solutions to enhance extreme environment tolerance. Green degradable design: Develop biomass aerogels based on enzymatic catalytic processes, reducing the full-lifecycle carbon footprint to one-third of traditional materials, while exploring chemical recycling and depolymerization technologies for polyester-based aerogels. These breakthroughs require deep integration of computational materials science (optimizing spatial distribution of flame retardants), in situ characterization (real-time analysis of structural evolution during thermal runaway via high-resolution microscopy), and bionic design, driving materials toward multifunctional integration, intelligent response, and full-chain green evolution, ultimately enabling large-scale application of high-safety, long-life, low-cost new energy battery systems.

### 3.2. Inorganic Aerogels and Their Composite Materials for Battery Thermal Runaway Protection

**Figure 10:**
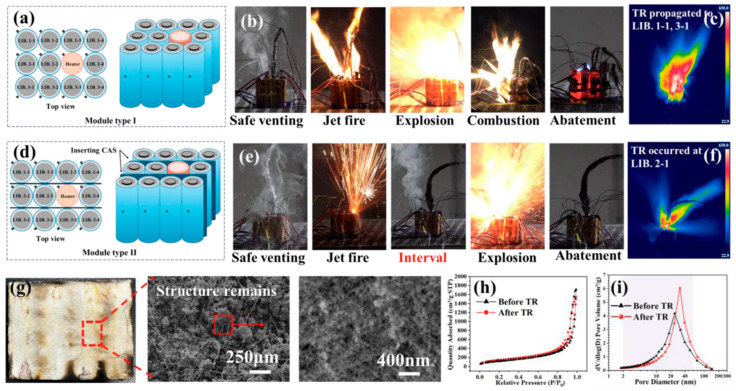
Thermal runaway propagation tests are designed to explore the blocking function of co-preform aerogel sheets in battery modules [138]. (**a**) Assembly of the LIB module; (**b**) Snapshots of TR propagation events at different stages; (**c**) Infrared image; (**d**) Assembly of the LIB module; (**e**) Snapshots of TR propagation events at different stages; (**f**) Infrared image; (**g**) Photos and scanning electron microscope images of CAS after TR test, showing that CA still maintains a porous structure; (**h**) Nitrogen adsorption-desorption curve; (**i**) Distribution of CAS pore diameters before and after TR test. Reproduced with permission from ref. [138]. Copyright 2023 Wiley-VCH.

Inorganic aerogels and their composite materials serve as a new generation of high-performance flame-retardant and heat-insulating materials [133], playing a crucial role in addressing the safety challenges of new energy batteries [134,135,136]. Currently, the main applications include silicon-based (SiO_2_) aerogels, metal oxide (such as boehmite, alumina) aerogels, and their composite systems [137]. Silicon-based aerogels, with their ultra-low thermal conductivity (0.014–0.022 W·m^−1^·K^−1^), high porosity (>90%), and nanoscale pore structure, can effectively block heat transfer, while metal oxide aerogels such as boehmite exhibit higher thermal stability (with a tolerance of >1000 °C) and self-extinguishing properties (with a limit oxygen index of up to 50%). To overcome the limitations of a single material, research focuses on composite design: for example, by introducing nano-boehmite sheets to enhance the SiO2 network, the composite material can have both low thermal conductivity and high crack resistance; graphene composite silicon-based aerogels utilize the rapid thermal diffusion ability of graphene and the heat-insulating properties of aerogels to work together, allowing the battery working temperature difference to be controlled within ±2 °C, significantly reducing the risk of thermal runaway. Xiao et al. reported a novel method, namely in situ supercritical separation (ISS), which utilizes internal equipment to fabricate co-preform aerogel sheets (CAS). The barrier function of CAS was verified through a series of experiments, which triggered thermal cracks by overusing heating. Therefore, by applying a 2-millimeter-thick CAS material between fully charged lithium-ion batteries with a maximum temperature of 836.2 degrees Celsius, the propagation of the thermal reaction was successfully inhibited, achieving a maximum temperature difference of 767 degrees Celsius between battery cells, as shown in Figure 10 [138].

Meanwhile, inorganic aerogel fiber mats, as a new generation of high-performance heat-insulating materials, are rapidly emerging in the field of new energy battery safety. Their unique nanoporous structure gives them ultra-low thermal conductivity at room temperature (0.017–0.025 W/(m·K)) and excellent high-temperature resistance, making them an ideal choice for solving the problem of lithium-ion battery thermal runaway. With the rapid development of electric vehicles and energy storage stations, the high energy density of battery packs has made the heat management challenge increasingly severe. Traditional organic flame-retardant materials often fail to meet the protection requirements in extreme thermal runaway environments above 800 °C. Inorganic aerogel fiber mats, with their inorganic nature and non-flammable characteristics, provide a new solution for fire isolation between battery modules. Inorganic aerogel fiber mats are mainly composed of silica aerogels and inorganic fiber substrates, and the fiber framework enhances the problem of mechanical brittleness of pure aerogels. Common types include ceramic fiber-reinforced composites, glass fiber composites, and ceramizable silicone rubber composites. These materials have excellent flexibility and cutability at room temperature and exhibit stable heat-insulating and flame-retardant properties at high temperatures. In response to the special requirements of new energy battery systems, the practicality research of inorganic aerogel fiber mats has shifted from simple heat insulation to multifunctional integration. The first priority is the optimization of spatial adaptability—the battery pack has a compact space, and the module gap is usually less than 5 mm, requiring the material to have both ultra-thin and efficient heat insulation properties. The matrix-type aerogel sheet design adopts a segmented strategy: individual membrane sheets are joined by hinge edges, allowing for flexible adjustment of the overall size and avoiding edge structure damage caused by cutting. More unique is its embedded positioning system—the positioning plug in the gap of the adhesive sheet and the flame-retardant dust-proof fabric are integrated to ensure that the membrane sheet group does not shift in the vehicle vibration environment. In the aspect of heat runaway interruption, researchers focus on the dual mechanisms of heat transfer path interruption and mass diffusion control. The low-radiation heat conduction property of aerogel fiber mats can delay the heat transfer between adjacent cells, giving the battery management system (BMS) a critical response time.

**Figure 11:**
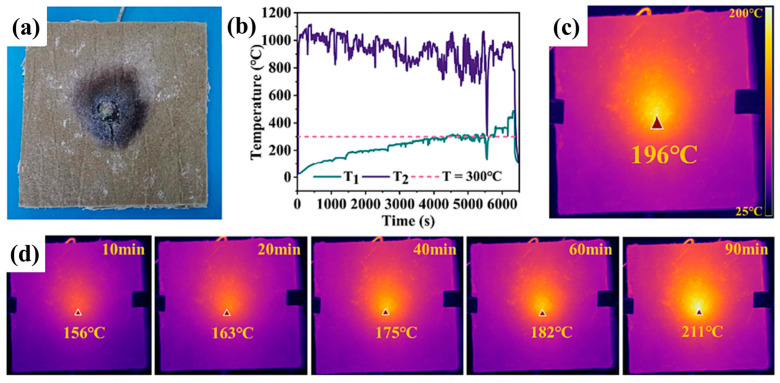
Test results of the thermal insulation of 10 mm thick basalt fiber mat/foam composite [139], (**a**) BFAC after heating; (**b**) The temperature T2 of the composite material surface and the temperature T1 inside the material during heating; (**c**) The temperature captured by the infrared camera on the backside of the steel plate when it fractures at 4500 seconds; (**d**) The temperature on the backside of the steel plate during heating. Reproduced with permission from ref. [139]. Copyright 2023 Elsevier.

According to the analysis results of battery power characteristics, after the cell of the battery undergoes thermal runaway, the surface temperature can reach above 800 °C, and the jet flame temperature can even reach above 1200 °C. Therefore, the heat-insulating material is required to have a long-term use temperature of 800 °C, and it needs to be able to withstand a high temperature of 1200 °C in the short term. The main types of fibers used in large-scale commercial applications of fiber-reinforced aerogels include pre-oxidized silk fibers, glass fibers, ceramic fibers, and basalt fibers, among others. Among them, pre-oxidized silk fibers and glass fibers have low usage temperatures and cannot be used at temperatures exceeding 800 °C. Ceramic fibers and SiO_2_ both have poor infrared radiation transparency and poor radiation heat transfer inhibition performance at high temperatures, which limits their high-temperature insulation performance. Ge et al. prepared basalt fiber felt/aerogel composites (BFAC) by making basalt fibers into a mat form and combining them with SiO_2_ aerogel composites and conducted thermal shielding tests and thermal stability analysis on them. The results showed that at 1000 °C, the cold surface temperature of 5 and 10 mm thick BFAC was less than 300 °C within 3000 and 5000 s, as shown in Figure 11 [139].

In terms of high-temperature fire resistance, recent studies have achieved performance breakthroughs through the synergistic use of multiple fire-resistant mechanisms. On the one hand, the inherent fire resistance is enhanced through nanostructure design. On the other hand, multiple-level protection is constructed by introducing functional components. From the perspective of the practicality of new energy batteries, inorganic aerogel materials demonstrate unique value in the “source-blocking” strategy for thermal runaway protection. However, these materials still face the risk of structural collapse under extreme conditions (such as ultra-high-temperature oxidation environments). In addition, the brittleness and interface compatibility of aerogels lead to easy powdering and shedding in the vibration environment of battery charge and discharge cycles, which restricts long-term reliability. Preparation methods and cost control are the key bottlenecks for industrialization. The traditional supercritical drying process, due to its complex equipment and high energy consumption, increases costs, while the breakthrough in atmospheric drying technology has significantly improved economic efficiency.

## 4. Conclusions and Prospects

### 4.1. Conclusions

This paper systematically reviews the latest advances in addressing thermal runaway challenges in high-energy-density battery systems through the application of aerogel-based thermal insulation and fireproof composites. Studies indicate that conventional flame-retardant materials fall short in providing sufficient thermal insulation and fire resistance under extreme thermal failure conditions. In contrast, aerogel materials leverage their unique nanoporous structure to deliver ultra-low thermal conductivity and exceptional fire resistance, showcasing irreplaceable technical advantages. Through innovations in material systems, process optimizations, and fiber–matrix compositing techniques, aerogel composites have made significant progress in overcoming limitations related to brittleness, cost, and scalability. Quantitative analyses affirm that their core engineering benefits in battery pack applications include effectively blocking the chain reaction of thermal runaway and preventing its propagation; substantially reducing system weight while compensating for added protection mass; and improving volumetric energy density at both module and system levels. As a result, aerogel composites have emerged as a highly promising revolutionary material for constructing the next generation of efficient and lightweight battery thermal management protection systems.

### 4.2. Prospects

**Figure 12:**
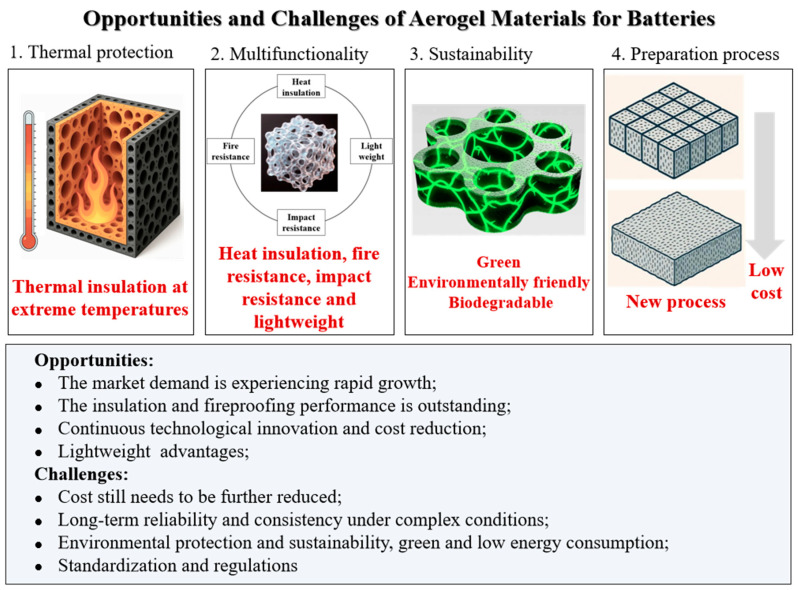
The opportunities and challenges for the future development of aerogel insulation and fireproof materials for batteries.

Although significant progress has been made in the application of aerogel composites in battery thermal protection, large-scale industrial application still faces challenges. Future research can focus on the directions for breakthroughs shown in Figure 12.

1. Development of low-cost and green preparation processes: Currently, the cost of aerogels, especially high-performance organic aerogels, remains relatively high. In the future, it is necessary to further develop large-scale preparation technologies with widely available raw materials, shortened process flows (such as complete atmospheric drying technology), and lower energy consumption to reduce the overall cost and meet the strict cost control requirements of the automotive industry. Comparing the current production process of aerogel, it can be seen that the production equipment and technical route of aerogel have shown a pattern of diversified development. Supercritical drying production lines can produce the most high-performance aerogel, but the high cost restricts its wide application. Atmospheric drying production lines have successfully realized low-cost, large-scale and green aerogel production through technological innovation, which has greatly promoted the industrialization process and market application of this material, making them the mainstream choice in the current civil and industrial fields. It is estimated that atmospheric drying technology is expected to reduce the production cost of aerogel by 20–40%. The production lines and surface functionalization equipment of various composite aerogel products meet the specific needs of the market for material morphology, functionality and application convenience.
2. Design of organic–inorganic hybrid composite structures: Pure inorganic or organic aerogels each have their advantages and disadvantages. In the future, efforts should be made to design organic–inorganic hybrid aerogels at the microscale or multi-layer composite structures at the macroscale. For example, by combining the flexibility and hydrophobicity of organic components with the extremely high temperature resistance of inorganic components, intelligent composite materials that are “rigid yet flexible” and integrate multiple functions (such as heat insulation, flame retardancy, buffering, and sound absorption) can be developed.
3. Research on full-scenario adaptability and long-term reliability: The internal environment of battery packs is complex, with conditions such as vibration, compression, and cold and hot cycles. It is necessary to deeply study the performance degradation laws and failure mechanisms of aerogel composites under long-term mechanical stress, wet heat aging, and electrolyte corrosion and establish a reliability evaluation system that matches the entire life cycle of batteries.
4. Standardization and integrated design: It is vital to promote the establishment of application standards for aerogel materials in the automotive battery field and standardize their performance testing and evaluation methods. At the same time, we must strengthen the integrated design of aerogels with battery pack structural components (such as Cell to Pack/Cell to Car, etc.) to maximize their protective value and space utilization efficiency from a system perspective.

In summary, aerogel composites provide a key material basis for resolving the contradiction between battery thermal safety and energy density improvement. Through continuous technological innovation and interdisciplinary cooperation, low-cost, high-performance, and multifunctional aerogel solutions will accelerate the development and industrialization of next-generation high-performance battery management systems, laying a solid foundation for the safety of new energy vehicles.

## Figures and Tables

**Table 1:** Performance comparison of heat insulation and fireproof materials for various types of automotive batteries.

Material Type	Thermal Conductivity (W/(m·K))	Temperature Resistance (°C)	Density (g/cm^3^)	Flame- Retardant Property	Cost	Advantages	Disadvantages	Main Application
Traditional foam [62,63,64,65,66]	0.02~0.04	≤300	0.03~0.05	Non-flammable, low smoke, with a fire resistance rating up to Class B1	Low	Low cost	Flammable, releases toxic gases, short lifespan	Early battery pack
Vacuum insulation panel [69,70,71,72]	<0.01	-	~0.2	Class A non-combustible	Relatively high	Ultra-low thermal conductivity	The dependence on maintaining a constant vacuum level over an extended period of time	Automobile power batteries
Polymer aerogels [44,45]	0.020–0.035	−196~500	0.10~0.30	Non-flammable, low smoke, with a fire resistance rating up to Class B1	High	Flexible, foldable, and high mechanical strength	Low temperature resistance and high cost	Military industry and high-end vehicle protection
Inorganic Aerogels [53,54,55,56,57,58]	~0.020	1200	0.1~0.2	Class A non-combustible, high limit oxygen index (LOI)	Relative high	Ultra-low thermal conductivity and high-temperature resistance	Brittle, low mechanical strength	Aerospace, energy, batteries
Inorganic aerogel composites [54,78,79,80]	0.015~0.025	650~1200	0.15~0.25	Class A non-combustible, and further enhance it by adding a fireproof and flame-retardant layer	Medium	Ultra-low thermal conductivity, lightweight	High brittleness and expensive cost	High-voltage fast-charging battery system

## Data Availability

No new data were created or analyzed in this study. Data sharing is not applicable to this article.
